# A sixteen-year epidemiological study of match injuries sustained during the women's Rugby World Cup (2010 to 2025)

**DOI:** 10.3389/fspor.2026.1812403

**Published:** 2026-07-27

**Authors:** Colin W. Fuller, Aileen Taylor

**Affiliations:** 1 Colin Fuller Consultancy Ltd, Sutton Bonington, United Kingdom; 2 Colin Fuller Consultancy Ltd, Nairobi, Kenya

**Keywords:** burden, concussion, incidence, knee ligament, nature, severity

## Abstract

**Purpose:**

Professionalisation of women's rugby has increased over the past ten years. The impact of this development on the incidence and nature of injuries sustained during international competitions has not been reported. This study aimed to provide anthropometric and epidemiological information relating to women's international Rugby-15s.

**Methods:**

Team medical staff, working with eighteen national teams taking part in five women's Rugby World Cups over the period 2010–2025, recorded players’ anthropometric and injury data. The observational methodology used in the study followed the international consensus statement for surveillance studies in elite rugby. Data relating to the incidence, severity, burden, nature and cause of injuries were collected.

**Results:**

The overall incidence of injury over the 16-year period was 45.2 injuries/1,000 player-match-hours with a mean severity of 48.4 days. Concussion (21.7%), knee-ligament (12.5%) and ankle-ligament (10.8%) injuries were the most common injuries sustained by backs and knee-ligament (17.2%), concussion (14.2%) and ankle-ligament (8.2%) injuries the most common for forwards. Knee-ligament injuries were responsible for 33.0% of all time-loss by backs and 43.2% of all time-loss by forwards.

**Discussion:**

The incidence of injury during the women's Rugby World Cup was significantly lower but the mean severity of the injuries significantly higher than values reported previously for the men's Rugby World Cup. Consequently, injury burden values during the women's and men's Rugby World Cups are similar. Knee-ligament and concussion injuries should be the focus of injury prevention initiatives in women's rugby, as they are the most common injuries and are responsible for the greatest time-loss.

## Introduction

Since rugby union became a professional sport in 1995, significant changes have taken place in the way the game is played at the elite level (1). These changes include the introduction of tactical player substitutions, increased ball-in-play time, players allowed to be lifted at lineouts, 20-minute red cards and the use of video-assistant-referees to support match officials’ on-field decisions. Many changes, however, have resulted from World Rugby's philosophy of ‘player welfare is the sport's number one priority’ (2). In this context, changes include the introduction of the crouch-bind-set protocol for scrum engagement, in-game player head injury assessments, instrumented mouthguards to record players’ head acceleration events and guidelines for players’ annual match and training exposure limits (3). The potential impacts of these game changes on player welfare are assessed through World Rugby's injury surveillance studies (ISS) (4–7), which monitor the incidence, nature and causes of match injuries. Routine ISS were introduced for international men's rugby competitions during the 2003 Rugby World Cup (8) and for international women's rugby during the 2006 Rugby World Cup (9). Comprehensive epidemiological information for international rugby has subsequently been gathered and published for men's Rugby-7s (10–12) and Rugby-15s (13–18) competitions. For women's rugby, Rugby-7s has been the focus (11, 19) with published epidemiological information for Rugby-15s limited to the 2006 (9) and 2010 (20) women's Rugby World Cups (WRWC) and the 2023 and 2024 WXV competitions (21).

As with all professional sports, in addition to the immediate, short-term consequences of injuries, there are possibilities that athletes may develop post-career health conditions related to injuries sustained during their playing careers. It has been reported, for example, that rugby players experience a higher prevalence of knee joint osteoarthritis post-career than athletes retiring from non-contact sports (22, 23). There have also been reports of a higher prevalence of neurodegenerative disease amongst retired rugby players, as a consequence of game-related head impacts (24–26), compared to the general population. Monitoring the incidences and causes of injuries that may lead to post-career health conditions is, therefore, an important aspect of World Rugby's ongoing ISS. In 2025, World Rugby, in conjunction with the International Rugby Players Association, agreed guidelines (27) for the implementation of annual match exposure limits (28) for elite players, with the aim of reducing their risk of developing adverse health conditions later in life.

Since professionalisation, most game developments in rugby have originated from and been based on the men's game. A 2025 review of injury reporting in women's rugby concluded that there is “*a dearth of injury surveillance research in women's versus men's rugby*” (29). Furthermore, the review reported that “*current literature fails to determine with confidence, women's specific risk factors, and if there are differences in injury surveillance and injury risk between men and women*” (29). The purpose of the present research paper is to address these concerns by reporting players’ anthropometric and match injury data collected during the five WRWC competitions that took place from 2010 to 2025. The 16-year timeframe covered by the study enables potential trends in players’ anthropometric data and the incidence, severity, nature and burden of injuries sustained during a period of professionalisation in women's rugby (30) to be reviewed.

## Materials and methods

Five WRWC competitions took place between 2010 and 2025 in England (2010), France (2014), Ireland (2017), New Zealand (2022), and England (2025). Twelve national teams competed in the 2010, 2014, 2017 and 2022 competitions and 16 teams in the 2025 competition. In total, nineteen countries, from six continents, participated in the five competitions. Eighteen of these countries, representing World Rugby's six Regional Associations (Asia Rugby: 3, Oceania Rugby: 3, Rugby Africa: 1, Rugby Americas North: 2, Rugby Europe: 8, Sudamerica Rugby: 1) provided players’ anthropometric and match injury data for this study. All aspects of this observational study were conducted in accordance with the definitions and protocols described in the World Rugby approved 2007 consensus statement for ISS in rugby union (31). Players’ anthropometric and injury data were recorded and reported prospectively by team medical staff at each competition. World Rugby's Institutional Ethics Committee approved the studies, as part of their ongoing ISS strategy. Players taking part in the competitions agreed to the collection of their anthropometric and injury data subject to the subsequent analysis and reporting of information being in a non-identifiable, generalised format.

### Data collection

Team medical staff recorded players’ anthropometric data (playing position; date of birth; body mass, Kg; stature, cm) just prior to the start of each competition. Players’ time-loss injuries, which were defined according to the rugby consensus statement (31) as ‘*an injury sustained during a WRWC match that prevented a player from taking a full part in all normal training activities and/or match play for more than one day following the day of injury*’, were reported by team medical staff. Injuries were categorised by body location and type together with an appropriate OSICS Code (32). Injury causation (contact, non-contact) and onset (acute, gradual) were reported together with the activity associated with the onset of injury. The match period when players were injured (0–20, 21–40+, 41–60, 61–80 + minutes) and whether players were removed from the game (immediately, later-in-game, not-at-all) were also recorded. Injury severity was based on the number of days a player was unable to undertake normal training and be available for match selection. For the few injuries that remained unresolved 90 days post-competition, severity was based on the injury diagnosis, treatment and rehabilitation received and a return-to-play prognosis provided by the injured players’ medical staff. Match injury burden values were defined as the product of match injury incidence and mean severity values (33–36). Match exposures (player-match-hours) were based on the assumption that 15 players (backs: 7; forwards: 8) per team were exposed for 80 min per game.

### Statistical analysis

Players’ anthropometric data were compared using unpaired t-tests (37). *Z*-tests were used to compare values of incidence, mean severity and proportions of injuries (37). Mann–Whitney U-tests were used to compare median severity values (37). Exact *p*-values are reported for all statistical tests with differences considered to be statistically significant if the *p* value was ≤0.050. Distributions of injury locations and types and match activities were compared across groups using the 95% confidence intervals (CI) associated with the parameter values; differences in values were considered statistically significant if the CIs did not overlap (37, 38).

Trends in players’ anthropometric data and injury incidence and burden values are reported based on linear regression analyses (slope, *p*-value). These trends include data from the five WRWC competitions that took place in the 16-year period from 2010 to 2025.

## Results

### Players’ anthropometric characteristics

**Figure 1:**
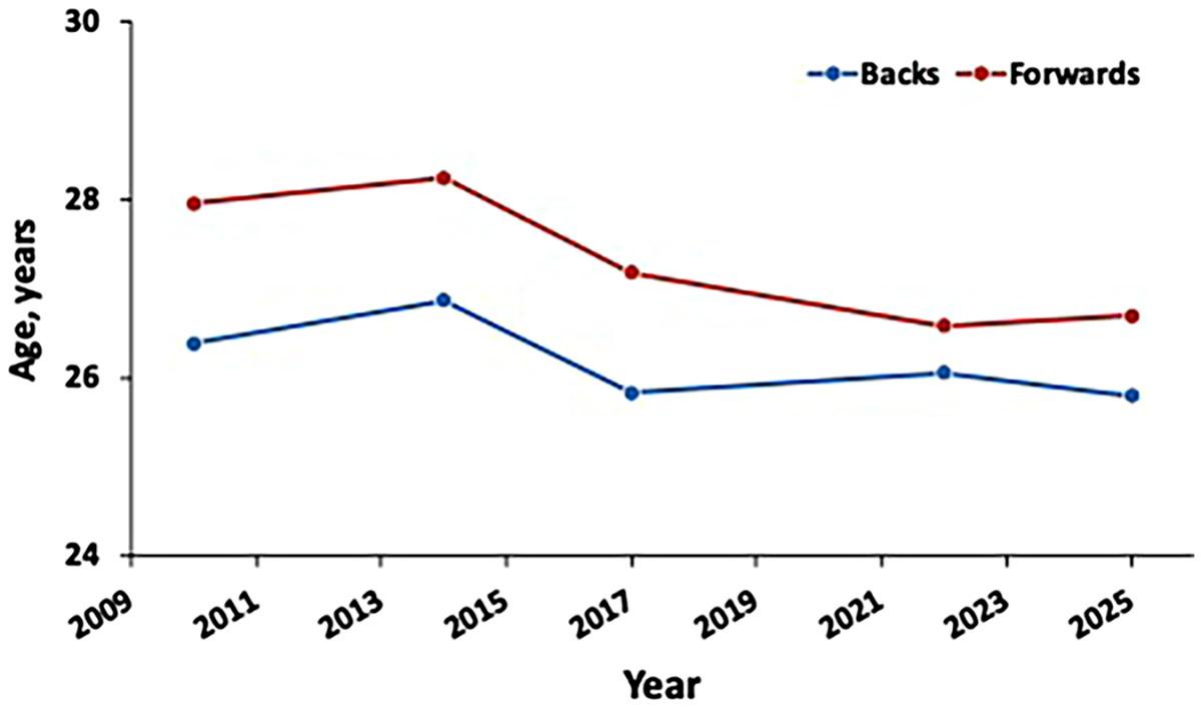
Trends in players’ age, as a function of playing position.

**Figure 2:**
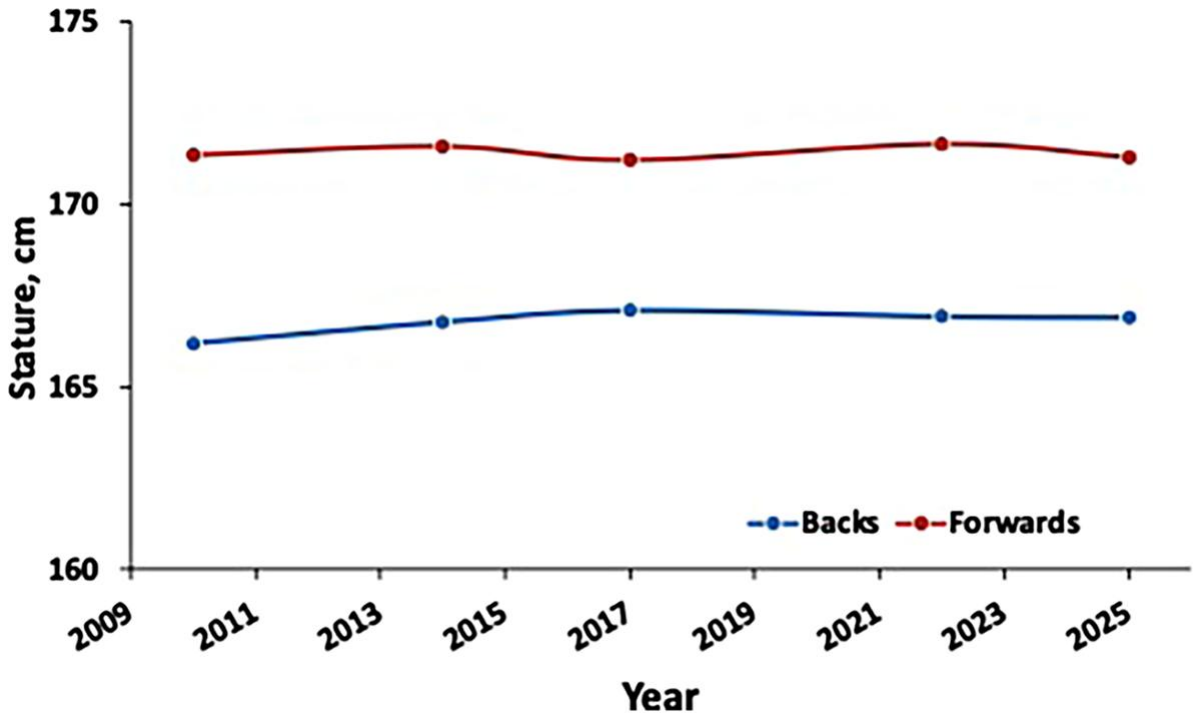
Trends in players’ stature, as a function of playing position.

**Figure 3:**
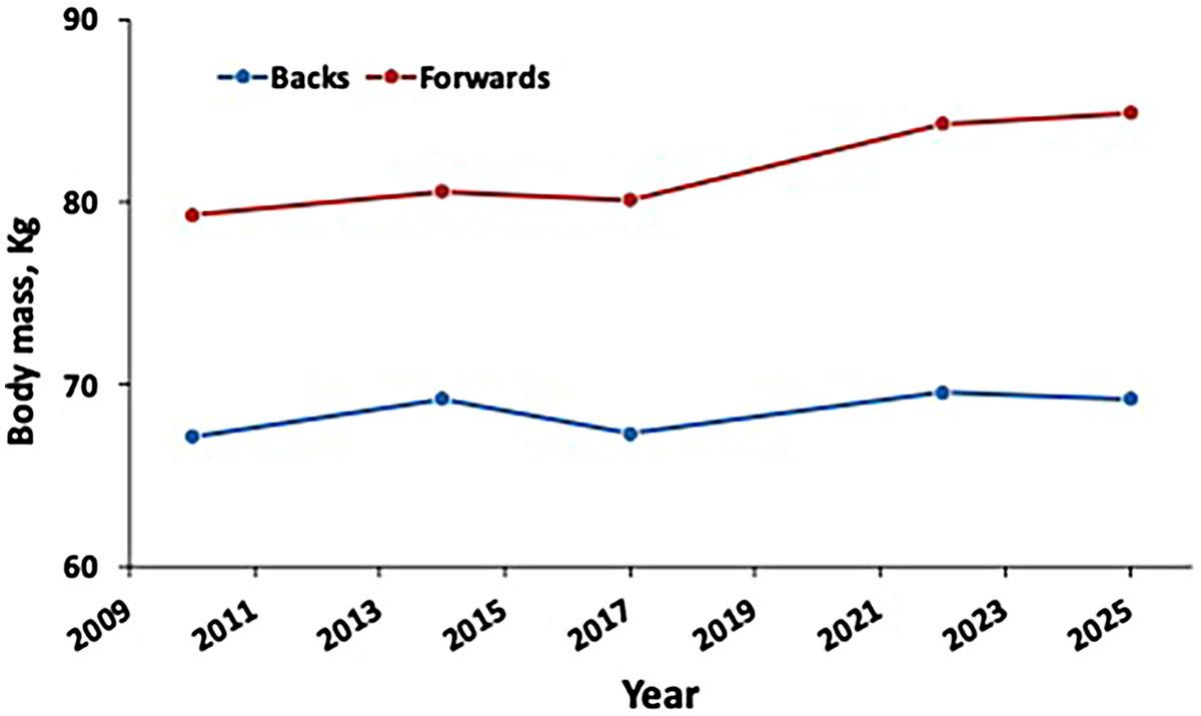
Trends in players’ body mass, as a function of playing position.

A total of 1,792 players (backs: 785; forwards: 1,007) were involved in the five WRWC competitions; Table 1 summarises the average age, stature and body mass of the players taking part in the five competitions. Forwards were significantly older (*p* < 0.001), taller (*p* < 0.001) and heavier (*p* < 0.001) than backs. Figures 1–3 show the trends in average age, stature and body mass of the backs and forwards competing in the 5 competitions. There have been small non-significant increasing trends in players’ stature over this period (backs: slope=+0.04 cm/year, *p* = 0.225; forwards: slope=<0.01, *p* = 0.995). There has been a statistically significant increasing trend in forwards’ body mass (slope=+0.40 Kg/year, *p* = 0.016) and a non-significant increasing trend in backs’ body mass (slope=+0.13 Kg/year, *p* = 0.232). Players experiencing the greatest increase in body mass were the front row forwards (81.9 Kg at the 2010 WRWC to 89.7 Kg at the 2025 WRWC). There has been a statistically significant decreasing trend in forwards’ age over the five competitions (slope=−0.11 years/year, *p* = 0.043) and a non-significant decreasing trend in the age of backs (slope=−0.05 years/year, *p* = 0.215) over these competitions.

**Table 1:** Anthropometric data for backs and forwards.

Measure	Mean (number of players, standard deviation)		
	Age	Stature	Body mass
**Backs**	**26.1** **(769, 4.2)**	**166.8** (**768, 5.9)**	**68.6** (**769, 7.6)**
Halves	26.3 (264, 4.3)	164.7 (264, 5.9)	66.6 (264, 7.4)
Inside backs	26.3 (208, 4.2)	168.1 (208, 5.7)	72.3 (208, 7.8)
Outside backs	25.9 (297, 3.9)	167.7 (296, 5.5)	67.7 (297, 6.6)
**Forwards**	**27.2** (**1,005, 4.4)**	**171.4** (**1,005, 6.6)**	**82.4** (**1,005, 10.9)**
Front row	27.2 (453, 4.4)	168.5 (453, 5.9)	86.7 (453, 12.0)
Second row	27.1 (237, 4.3)	177.5 (237, 5.0)	81.6 (237, 8.1)
Back row	27.2 (315, 4.4)	171.0 (315, 5.4)	76.9 (315, 8.2)
**All players**	**26.7** (**1,774, 4.3)**	**169.4** (**1,773, 6.7)**	**76.4** (**1,774, 11.8)**

Injured players were, on average, significantly (*p* = 0.031) lighter (74.7 Kg) than the overall sample population (76.4Kg). There were no significant differences, however, in the average stature (169.0 cm) or age (26.7 years) of injured players compared to the sample population (stature:169.4 Kg, *p* = 0.373; age: 26.7 years, *p* = 1.000).

### Match injuries

The numbers of match injuries sustained by backs, forwards and all players during the five WRWC competitions, the associated match exposures and the resultant incidences of match injuries are shown in Table 2. Fourteen of the injuries reported (backs: 5, forwards 9) were recorded as recurrences (39). No catastrophic or career-ending injuries were reported during the five WRWC competitions. The difference between the incidence values for backs and forwards is not statistically significant (*p* = 0.849). For players sustaining an injury of any type, 40.7% were removed from play immediately (backs: 39.5%, forwards: 41.8%), 27.3% were removed later (backs: 26.1%, forwards: 28.4%) and 32.0% remained on the pitch and completed the game (backs: 34.5%, forwards: 29.9%). Considering concussion injuries alone, 72.7% of the players (backs: 64.0%, forwards: 84.2%) were removed from play immediately, 18.2% (backs: 20.0%, forwards: 15.8%) were removed later in the game and 9.1% (backs: 16.0%, forwards: 0.0%) remained on the pitch until the end of the game. The difference between the proportion of concussed players removed immediately from play (72.7%) and the proportion of players removed immediately when sustaining any other type of injury (34.0%) was statistically significant (*p* < 0.001).

**Table 2:** Number, exposure (player-hours), incidence (injuries/1,000 player-match-hours, 95% CI), mean and median severity (days, 95% CI) and injury burden (days-absence/1,000 player-match-hours, 95% CI) for match injuries sustained by backs and forwards.

Measure	Backs	Forwards	ALL players
Injuries	120	134	254
Exposure	2,623	2,997	5,620
Incidence	45.8 (38.3–54.7)	44.7 (37.7–53.0)	45.2 (40.0–51.1)
Severity, mean	50.2 (35.1–65.3)	46.7 (35.2–58.1)	48.4 (39.0–57.7)
Severity, median	10.5 (7.0–17.0)	13.0 (9.0–25.0)	11.0 (9.0–17.0)
Injury burden	2,298 (1,921–2,748)	2,087 (1,762–2,472)	2,185 (1,932–2,471)

Table 2 also shows the mean and median injury severities and the injury burden values for backs, forwards and all players. The 254 injuries sustained across the five competitions led to a total of 12,281 player-days-absence (backs: 6,027, forwards: 6,254). Although the mean injury severity value for backs is higher than that recorded for forwards, the difference is not statistically significant (*p* = 0.719). The median injury severity value for forwards was higher than that of backs; this difference was also not statistically significant (*p* = 0.613). The difference in the injury burden values for backs and forwards is not statistically significant (*p* = 0.441).

**Figure 4:**
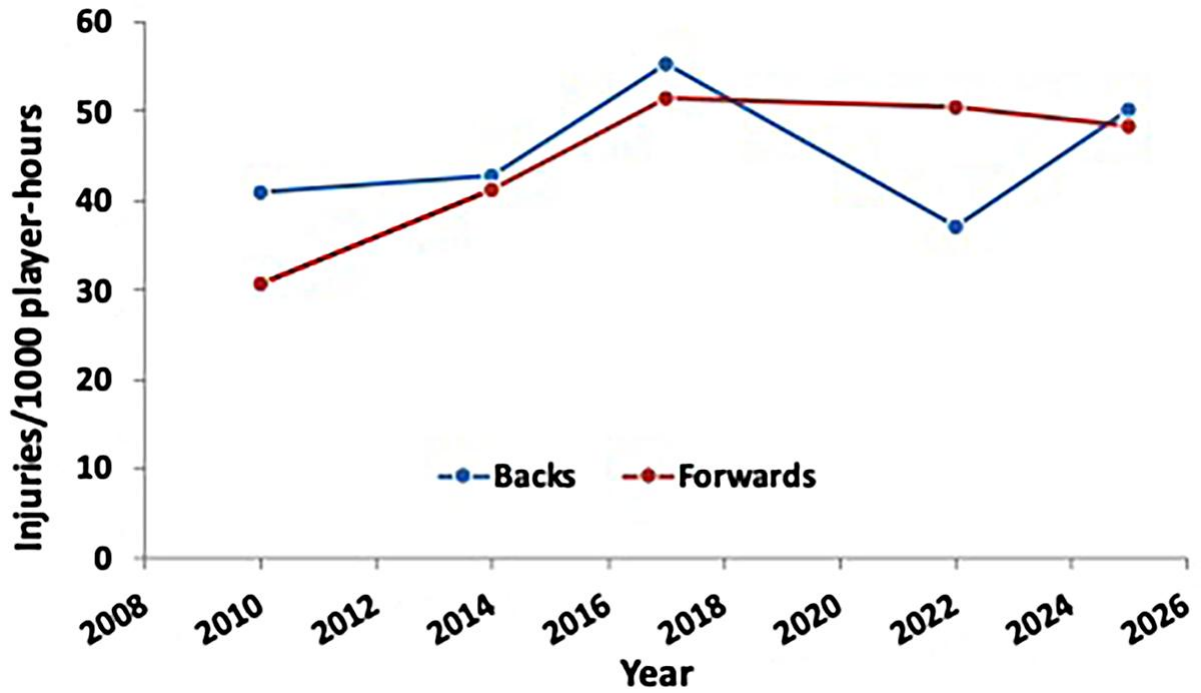
Trends in injury incidence, as a function of playing position.

**Figure 5:**
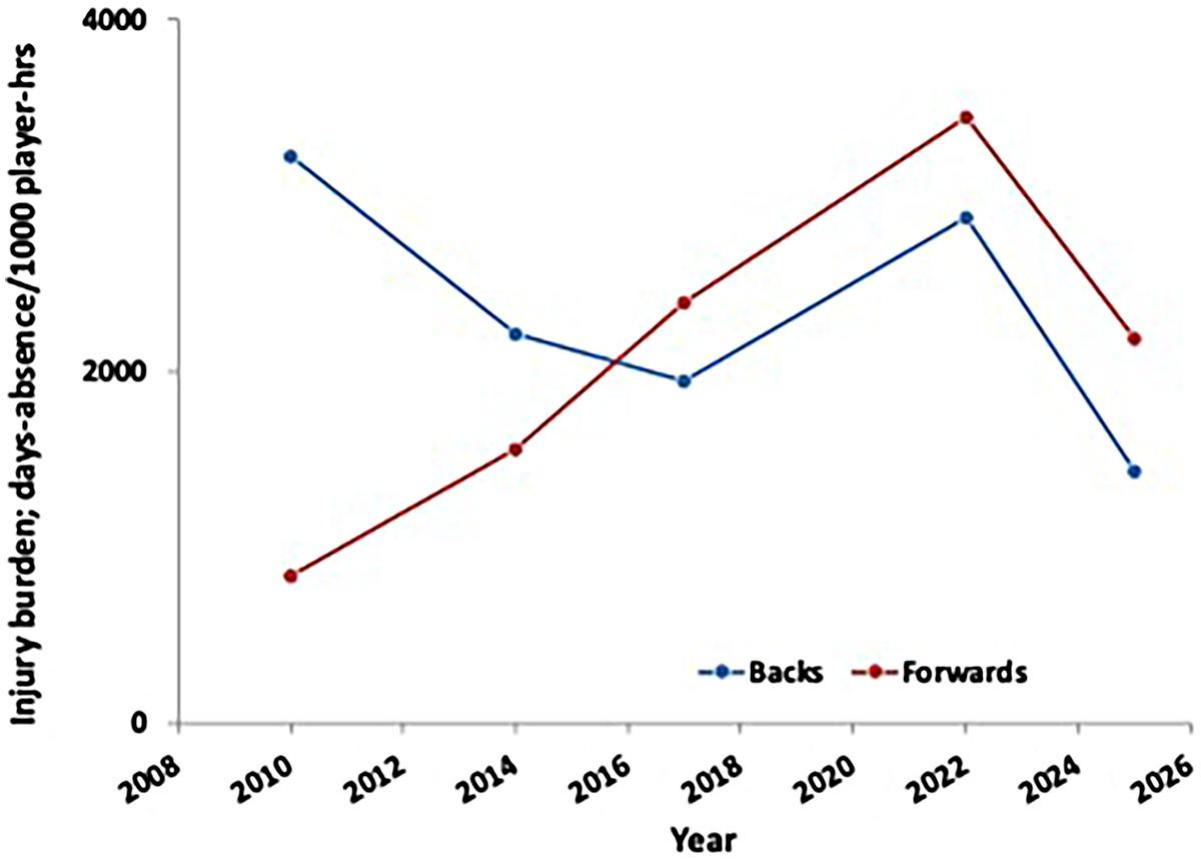
Trends in injury burden, as a function of playing position.

Figure 4 shows the trends in match injury incidence and Figure 5 the trends in injury burden for backs and forwards, over the 5 competitions. There were no statistically significant trends in either the incidence (backs: slope=+0.25 injuries/1000 player-hours/year, *p* = 0.763; forwards: slope=+1.2, *p* = 0.105) or burden of injury (backs: slope=−71 days-absence/1000 player-hours/year, *p* = 0.287; forwards: slope=+124, *p* = 0.132) values.

The locations and types of injuries sustained during the five WRWC competitions are reported in Table 3. Based on the 95% CI values for the various locations and types of injury, the only statistically significant differences between backs and forwards relate to the small percentages of elbow, lower back and hip/groin injuries sustained by forwards. For backs, concussion (21.7%; 95% CI: 14.3–29.0), knee-ligament (12.5%; 95% CI: 6.6–18.4), ankle-ligament (10.8%; 5.3–16.4), shoulder-ligament (5.0%; 95% CI: 1.1–8.9) and lower leg-muscle strains (5.0%; 95% CI: 1.1–8.9) were the five most common injuries sustained; these injuries accounted for 55.0% of all injuries sustained by backs. For forwards, knee-ligament (17.2%; 95% CI: 10.8–23.5), concussion (14.2%; 95% CI: 8.3–20.1), ankle-ligament (8.2%; 95% CI: 3.6–12.9), hand-fracture (6.7%; 95% CI: 2.5–11.0) and shoulder-ligament (6.0%; 95% CI: 2.0–10.0) injuries were the five most common injuries sustained; these injuries accounted for 52.2% of all injuries sustained by forwards.

**Table 3:** Locations and types of match injury sustained by backs and forwards.

Injury location/type	% (95% confidence interval)		
	Backs	Forwards	ALL players
INJURY LOCATION -			
**Head/neck**	**30.0** **(21.8–38.2)**	**21.6** (**14.7–28.6)**	**25.6** (**20.2–31.0)**
Head/face	28.3 (20.3–36.4)	18.7 (12.1–25.3)	23.2 (18.0–28.4)
Neck/cerv^l^ spine	1.7 (0–4.0)	3.0 (0.1–5.9)	2.4 (0.5–4.2)
**Upper limbs**	**20.0** (**12.8–27.2)**	**25.4** (**18.0–32.7)**	**22.8** (**17.7–28.0)**
Shoulder/clavicle	12.5 (6.6–18.4)	11.9 (6.4–17.4)	12.2 (8.2–16.2)
Upper arm	1.7 (0–4.0)	0.0 (-)	0.8 (0–1.9)
Elbow	0.0 (-)	3.0 (0.1–5.9)	1.6 (0–3.1)
Forearm	0.8 (0–2.5)	0.0 (-)	0.4 (0–1.2)
Wrist/hand	5.0 (1.1–8.9)	10.4 (5.3–15.6)	7.9 (4.6–11.2)
**Trunk**	**3.3** (**0.1–6.5)**	**6.7** (**2.5–11.0)**	**5.1** (**2.4–7.8)**
Ribs/upper back	0.8 (0–2.5)	0.7 (0–2.2)	0.8 (0–1.9)
Abdomen	2.5 (0–5.3)	0.0 (-)	1.2 (0–2.5)
Lower back	0.0 (-)	5.2 (1.5–9.0)	2.8 (0.7–4.8)
Sacrum/pelvis	0.0 (-)	0.7 (0–2.2)	0.4 (0–1.2)
**Lower limbs**	**46.7** (**37.7–55.6)**	**46.3** (**37.8–54.7)**	**46.5** (**40.3–52.6)**
Hip/groin	0.0 (-)	3.0 (0.1–5.9)	1.6 (0–3.1)
Thigh, anterior	4.2 (0.6–7.7)	1.5 (0–3.5)	2.8 (0.7–4.8)
Thigh, posterior	3.3 (0.1–6.5)	0.7 (0–2.2)	2.0 (0.3–3.7)
Knee	18.3 (11.4–25.3)	23.1 (16.0–30.3)	20.9 (15.9–25.9)
Lower leg	7.5 (2.8–12.2)	6.0 (2.0–10.0)	6.7 (3.6–9.8)
Ankle	11.7 (5.9–17.4)	9.7 (4.7–14.7)	10.6 (6.8–14.4)
Foot/toe	1.7 (0–4.0)	2.2 (0–4.7)	2.0 (0.3–3.7)
INJURY TYPE -			
**Bone**	**7.5** (**2.8–12.2)**	**14.9** (**8.9–21.0)**	**11.4** (**7.5–15.3)**
Fracture	7.5 (2.8–12.2)	13.4 (7.7–19.2)	10.6 (6.8–14.4)
Other bone	0.0 (-)	1.5 (0–3.5)	0.8 (0–1.9)
**C/PNS**	21.7 (14.3–29.0)	**14.9** (**8.9–21.0)**	**18.1** (**13.4–22.8)**
Concussion	21.7 (14.3–29.0)	14.2 (8.3–20.1)	17.7 (13.0–22.4)
Nerve	0.0 (-)	0.7 (0–2.2)	0.4 (0–1.2)
**Joint (non-bone)/lig** ^t^	**39.2** (**30.4–47.9)**	**48.5** (**40.0–57.0)**	**44.1** (**38.0–50.2)**
Dislocation/sublux^n^	2.5 (0–5.3	3.0 (0.1–5.9)	2.8 (0.7–4.8)
Lesion meniscus	2.5 (0–5.3	6.7 (2.5–11.0)	4.7 (2.1–7.3)
Sprain/ligament	34.2 (25.7–42.7)	37.3 (29.1–45.5)	35.8 (29.9–41.7)
Other	0.0 (-)	1.5 (0–3.5)	0.8 (0–1.9)
**Muscle/tendon**	**27.5** (**19.5–35.5)**	**17.9** (**11.4–24.4)**	**22.4** (**17.3**–**27.6)**
Haematoma/etc	8.3 (3.4–13.3)	8.2 (3.6–12.9)	8.3 (4.9–11.7)
Muscle rupture/etc	15.0 (8.6–21.4)	6.0 (2.0–10.0)	10.2 (6.5–14.0)
Tendon injury/etc	4.2 (0.6–7.7)	3.7 (0.5–6.9)	3.9 (1.5–6.3)
**Skin**	**1.7** (**0–4.0)**	**0.7** (**0–2.2)**	**1.2** (**0–2.5)**
Laceration	1.7 (0–4.0)	0.7 (0–2.2)	1.2 (0–2.5)
**Other injuries**	**2.5** (**0–5.3)**	**3.0** (**0.1–5.9)**	**2.8** (**0.7–4.8)**

C/PNS, central and peripheral nervous system.

In terms of the knee-ligament injuries reported above, 39.5% of these related to the anterior cruciate ligament (backs: 46.7%; forwards: 34.8%), 50.0% to the medial collateral ligament (backs: 40.0%; forwards: 56.5%) and 7.9% to the posterolateral ligament complex (backs: 13.3%; forwards: 4.3%). The mean severities of the knee-ligament injuries were anterior cruciate ligament 234 days (backs: 258; forwards: 213), medial collateral ligament 57 days (backs: 24; forwards: 73) and posterolateral ligament complex 27 days (backs: 22; forwards: 36).

Regarding injury burden, knee-ligament (33.0%; 95% CI: 24.6–41.4), concussion (10.9%; 95% CI: 5.3–16.5), shoulder-ligament (7.8%; 95% CI: 3.0–12.6), shoulder-dislocation (6.7%; 95% CI: 2.2–11.2) and knee-meniscal (6.2%; 95% CI: 1.9–10.5) injuries accounted for 64.6% of all injury-related time-loss by backs. For forwards, knee-ligament (43.2%; 95% CI: 34.8–51.6), lower leg fracture (8.7%; 95% CI: 3.9–13.5), hand fracture (7.0%; 95% CI: 2.7–11.3), concussion (6.0%; 95% CI: 2.0–10.0) and knee fracture (4.7%; 95% CI: 1.1−8.2) injuries accounted for 69.5% of all time-lost.

### Injury risk factors

In terms of playing position, the incidence of injury was highest for back-row forwards (54.3 injuries/1000 player-hours), inside-backs (50.7 injuries/1000 player-hours) and outside-backs (45.4 injuries/1000 player-hours). Injury burden was highest for back-row forwards (2,780 days-absence/1000 player-hours), inside-backs (2,381 days-absence/1000 player-hours) and scrum-half/fly-half (2,377 days-absence/1000 player-hours). The majority (70.5%) of concussions were sustained by inside-backs (27.3%), back-row forwards (22.7%) and outside backs (20.5%); whereas, the majority of knee-ligament injuries (68.5%) were sustained by back-row forwards (26.3%), scrum-half/fly-half (21.1%) and front-row forwards (21.1%).

Significantly more injuries were sustained in the second half of matches for both backs (first half: 38.3%; second half: 61.7%; *p* = 0.012) and forwards (first half: 40.5%; second half: 59.5%; *p* = 0.029). Furthermore, a third of all injuries (backs: 37.4%; forwards: 29.8%) were sustained in the fourth quarter of matches and were responsible for 36.4% of all time-loss (backs: 43.2%; forwards: 29.9%). Over sixty per cent (backs: 68.8%; forwards: 50.0%) of all muscle rupture/strain injuries and 100% of non-contact, lower leg muscle strains were sustained in the fourth quarter of matches.

There was no significant difference (*p* = 0.322) in the nature of injury onset between backs (acute: 96.7%, 95% CI: 93.5–99.9%; gradual onset: 3.3%, 95% CI: 0.1%–6.5%) and forwards (acute: 94.0%, 95% CI: 90.0–98.0%; gradual onset: 6.0%, 95% CI: 2.0–10.0%). Overall, the majority of injuries (87.1%) resulted from contact match activities; forwards (contact: 91.6%, 95% CI: 86.9–96.4%; gradual onset: 8.4%, 95% CI: 3.6–13.1%), however, sustained significantly (*p* = 0.027) more injuries as a consequence of contact events than backs (contact: 82.1%, 95% CI: 75.1–89.0%; non-contact: 17.9%, 95% CI: 11.0–24.9%). Table 4 shows that being-tackled (33.3%), tackling (19.8%) and collision (16.2%) were the match activities most often associated with injuries sustained by backs and being-tackled (27.2%), collision (21.6%) and tackling (20.0%) the most often for forwards. For concussions, tackling (36.0%), being-tackled (24.0%) and collisions (24.0%) were the activities most often associated with backs’ injuries and collisions (42.1%), being-tackled (26.3%) and tackling (21.1%) the most often for forwards’ injuries. For knee-ligament injuries, being-tackled (46.7%), running (20.0%) and tackling (13.3%) were most often associated with injury for backs while being-tackled (36.4%), collisions (31.8%) and tackling (13.6%) were most often associated with injuries for forwards.

**Table 4:** Match activity associated with injuries sustained by backs and forwards.

Match activity	% (95% confidence interval)		
	Backs	Forwards	ALL players
Collision	16.2 (9.4–23.1)	21.6 (14.4–28.8)	19.1 (14.1–24.1)
Kicking	0.0 (-)	0.0 (-)	0.0 (-)
Lineout	0.0 (-)	1.6 (0–3.8)	0.8 (0–2.0)
Maul	0.9 (0–2.7)	3.2 (0.1–6.3)	2.1 (0.3–4.0)
Ruck	9.9 (4.4–15.5)	13.6 (7.6–19.6)	11.9 (7.7–16.0)
Running	15.3 (8.6–22.0)	4.8 (1.1–8.5)	9.7 (6.0–13.5)
Scrum	0.9 (0–2.7)	4.8 (1.1–8.5)	3.0 (0.8–5.1)
Tackled	33.3 (24.6–42.1)	27.2 (19.4–35.0)	30.1 (24.2–35.9)
Tackling	19.8 (12.4–27.2)	20.0 (13.0–27.0)	19.9 (14.8–25.0)
Other	3.6 (0.1–7.1)	3.2 (0.1–6.3)	3.4 (1.1–5.7)

## Discussion

This observational study provides a comprehensive overview of international female rugby players’ anthropometric and match injury data. The range and number of national teams involved in the study mean that the information provides a worldwide perspective on the risk of match injury in international women's rugby.

Over the 2010–2025 period (Figure 3), there was a 2.0 Kg increase in the body mass of backs (67.2–69.2 Kg) and a 5.1 Kg increase for forwards (79.3–84.9 Kg); the forwards’ increase was greatly influenced by the 7.8 Kg increase in the body mass of front-row forwards. WRWC forwards were, on average, 13.8 Kg heavier than backs (Table 1), which compares to a 20.6 Kg difference between forwards and backs reported for the men's 2019 RWC (18). Although the difference between forwards and backs, in terms of absolute body mass, is much larger for male players, the differences, in percentage terms, are similar for men's (22.6%) and women's (20.1%) competitions. The players’ stature and body mass recorded at the 2022 and 2025 WRWC (Figures 2, 3) are similar to the values reported previously in the epidemiological study of the women's 2023 and 2024 WXV competitions (21). Higher body mass, which increases players’ momentum and forces during match contact events, provides a competitive advantage for players during contact-based activities, such as tackles, collisions, rucks, mauls and scrums (40, 41). Increased impact forces during contact events could, however, increase the injury risk for players.

The incidence of injury at the five WRWC competitions (Table 2) is similar to the 49.5 injuries/1000 player match-hours reported previously for the 2023 and 2024 WXV competitions (21). The incidence for the WRWC is, however, significantly (*p* < 0.001) lower than that reported for the 2007–2019 men's RWCs (13, 15, 16, 18). Players were significantly more likely to be injured in the second half of matches, which is a situation often reported in both men's and women's Rugby-7s (12, 19) and Rugby-15s (13, 15–18) competitions. This situation is normally attributed to player fatigue, as fatigue adversely impacts players’ physical and cognitive abilities. Late-game fatigue amongst players is normally addressed through coach-driven, player-specific conditioning and match-day substitution strategies. It is also possible that players are prepared to take greater risks in the final stages of games in order to achieve a favourable match result.

The mean and median severity values recorded during WRWC are similar to those reported (21) for the WXV competition (mean: 42.2 days; median: 16.0 days). The mean severity values recorded for the WRWC are, however, nearly twice those reported for the men's RWC (13, 15, 16, 18), which is mainly a consequence of the greater propensity for women to sustain knee-ligament injuries, in particular high severity anterior cruciate ligament injuries (42). Although the overall incidence of injury sustained in the WRWC is half that reported for the men's RWC, the higher mean injury severity in the WRWC, results in similar injury burden values being recorded for the women's and men's RWCs (13, 15, 16, 18).

There were no meaningful differences in injury locations between the WRWC results and the 2023 and 2024 WXV results (21). The head/face (23.2%) and knee (20.9%) locations were the two most common injury sites during WRWC, which are similar to those reported for the WXV competitions (head/face: 22.8%; knee: 22.8%). As discussed above, the proportion of knee injuries sustained in the WRWC is much higher than that reported for the men's RWC (13, 15, 16, 18). Ligament injuries (35.8%) and concussions (17.7%) were the most common injury types reported during WRWC, which are again similar to the results reported for the WXV competitions (ligament: 35.6%; concussion: 14.9%) (21). The proportion of ligament injuries sustained during the WRWC is also much higher than that reported for the men's RWCs (13, 15, 16, 18).

The tackle was associated with fifty per cent (being-tackled: 30%; tackling: 20%) of all injuries sustained by players in the WRWC competitions (Table 4); however, this is not unexpected, as tackles are by far the most common match activity in rugby (43). While tackling was most often associated with concussions sustained by backs. collisions were most often associated with forwards’ concussions. For knee-ligament injuries, being-tackled was most often associated with these injuries for both backs and forwards.

### Injury prevention priorities

Differences in the nature and aetiology of injuries sustained by backs and forwards in women's and men's rugby emphasise that injury prevention initiatives must be both sex-specific and playing position-specific. Adopting the same, general-purpose injury prevention strategies across all players and all forms of rugby are unlikely to be as beneficial or cost effective as gender-specific approaches (34, 44). This study, which covers five WRWC competitions, together with results reported previously from a 2-year study of the women's WXV competition (21) identify concussion and knee-ligament injuries as the two highest priority player welfare issues for elite women's rugby. It has been reported that the risk factors associated with concussion (45, 46) and ACL (42, 47) injuries are not the same for female and male athletes and injury prevention strategies should, therefore, be gender-specific. From a short-term perspective, concussions are the most frequent injury sustained and knee-ligament injuries the cause of the greatest loss of time; consequently, these two injuries will have the greatest impact on player availability and hence team performance. There are also general concerns in both women's and men's rugby about these two injuries from a long-term, risk-management perspective, as potential links have been reported between concussion and neurodegenerative diseases such as dementia and Alzheimer's disease (25, 48), and between knee-ligament injuries and osteoarthritis in knee joints (49, 50).

Although measuring head impact forces during contact events is, intuitively, a simple injury prevention approach for concussion research, the links between the magnitude, frequency and nature of head impact events, concussions and long-term neurodegenerative diseases are complex and not yet clearly understood (51, 52). In addition, the implementation of annual player-exposure limits may be beneficial for players but the evidence-base for the limits proposed is not well-founded, at present (28). It remains important, therefore, to identify and understand the individual and synergistic impacts of risk factors, such as players’ physiological characteristics and injury history and the biomechanical nature of player contact events in order to identify the most effective injury prevention strategies for women's rugby (51, 52).

The strength of this epidemiological study is that all anthropometric and injury data were recorded by team medical staff, according to the requirements of the international consensus statement for rugby injury surveillance studies. The data reported cover teams from eighteen countries competing at five WRWCs over a sixteen-year period. The study, therefore, provides wide-ranging and comprehensive information about the incidence, severity, nature and causes of match injuries sustained in international women's rugby. The data reported also provides a benchmark of injury risks in women's elite rugby, against which injury prevention initiatives can be assessed. In particular, the data included in this report addresses the concern that there is a lack of epidemiological information about injuries sustained in women's international rugby compared to the level of information available for men's international rugby (29).

## Data Availability

The datasets presented in this article are not readily available because of the confidential nature of players’ personal medical information. Requests to access datasets are not applicable.
